# Satisfaction Levels and Factors Influencing Satisfaction With Use of a Social App for Neonatal and Pediatric Patient Transfer Information Systems: A Questionnaire Study Among Doctors

**DOI:** 10.2196/medinform.5984

**Published:** 2016-08-04

**Authors:** Iee Choi, Jin Kyu Kim, Sun Jun Kim, Soo Chul Cho, Il Nyeo Kim

**Affiliations:** ^1^Research Institute of Clinical Medicine of Chonbuk National University and Biomedical Research Institute of Chonbuk National University HospitalDepartment of PediatricsChonbuk National University HospitalJeonju, KoreaRepublic Of Korea

**Keywords:** social media, personal satisfaction, information systems, patient transfer

## Abstract

**Background:**

The treatment of neonatal and pediatric patients is limited to certain medical institutions depending on treatment difficulty. Effective patient transfers are necessary in situations where there are limited medical resources. In South Korea, the government has made a considerable effort to establish patient transfer systems using various means, such as websites, telephone, and so forth. However, in reality, the effort has not yet been effective.

**Objective:**

In this study, we ran a patient transfer information system using a social app for effective patient transfer. We analyzed the results, satisfaction levels, and the factors influencing satisfaction.

**Methods:**

Naver Band is a social app and mobile community application which in Korea is more popular than Facebook. It facilitates group communication. Using Naver Band, two systems were created: one by the Neonatal Intensive Care Unit and the other by the Department of Pediatrics at Chonbuk National University Children's Hospital, South Korea. The information necessary for patient transfers was provided to participating obstetricians (n=51) and pediatricians (n=90). We conducted a survey to evaluate the systems and reviewed the results retrospectively.

**Results:**

The number of patients transferred was reported to increase by 65% (26/40) obstetricians and 40% (23/57) pediatricians. The time taken for transfers was reported to decrease by 72% (29/40) obstetricians and 59% (34/57) pediatricians. Satisfaction was indicated by 83% (33/40) obstetricians and 89% (51/57) pediatricians. Regarding factors influencing satisfaction, the obstetricians reported communication with doctors in charge (*P*=.03) and time reduction during transfers (*P*=.02), whereas the pediatricians indicated review of the diagnosis and treatment of transferred patients (*P*=.01) and the time reduction during transfers (*P*=.007).

**Conclusions:**

The users were highly satisfied and different users indicated different factors of satisfaction. This finding implies that users’ requirements should be accommodated in future developments of patient transfer information systems.

## Introduction

The treatment of neonatal and pediatric patients in South Korea is limited to certain types of medical institutions depending on disease specificity, patient severity, and treatment difficulty. The amount of medical resources available varies greatly from region to region, with obvious differences in medical infrastructure and administration quality. It is necessary for each region to be equipped with highly trained medical professionals and competent medical facilities, but the availability of such resources is often limited [1-2]. Neonatal and pediatric patients, in particular, are frequently found in emergencies requiring immediate medical attention. When they are transferred from primary or secondary hospitals to tertiary hospitals, a considerable amount of time is often spent locating and identifying available hospital resources, causing significant treatment delays [3-6]. To address this issue, the Emergency Medical Service Act has been enacted in South Korea to strengthen the medical infrastructure. The government has taken the initiative of establishing a National Emergency Medical Center and providing the relevant medical information. Nevertheless, due to information inaccuracy and functional limitations, government authorities and medical professionals have begun to discuss a more efficient emergency medical information system [4].

For the efficient transfer of emergency patients, the emergency medical information provided must be easily accessible and accurate. To this end, social media are perceived as important platforms where users can easily access and share various information. Social media are Web-based services that allow users to form interpersonal networks and to use the networks to connect and communicate with new people [7,8]. The widespread use of mobile phones has enabled real-time communication on social media, and they are currently used in numerous fields due to the efficiency of their information-sharing capabilities. Social media are also widely used in medicine. Notable users include the Centers for Disease Control and Prevention, World Health Organization, and American Public Health Association, which use social media for their information sharing and communication efforts. There are ongoing studies into the use of social media in the medical field and its effectiveness in the United States and other countries [9-13]. In this study, we aimed to develop a model for using the real-time information-sharing function of social media as a patient transfer system. We used a social media platform to create and run a neonatal and pediatric patient transfer information system for obstetric and pediatric physicians in the Jeollabuk-do region of South Korea. We also conducted a questionnaire-based survey to assess the satisfaction with the system and to identify the factors related to satisfaction. We then used the data to identify areas requiring improvement to establish more effective patient transfer information systems in the future.

## Methods

### Study Design and Participants

The Neonatal Intensive Care Unit and Department of Pediatrics of Chonbuk National University Children's Hospital ran a neonatal and pediatric patient transfer information system (hereinafter, “the Bands”) using Naver Band, which is a closed-type social network service developed by the Internet portal Naver. The Neonatal Intensive Care Unit Band (hereinafter, “NICU Band”) was opened to obstetric physicians since August 2013 and the Department of Pediatrics Band (hereinafter, “DP Band”) was opened to pediatric physicians since November 2014.

The main operators of the NICU Band were the supervising professors and nurse practitioners in the NICU. The nurse practitioners provided daily notifications of the availability of beds and mechanical ventilation equipment, which are essential to patient transfers, so that local obstetricians could take necessary actions based on the information. As most of the neonatal patients transferred to the NICU are in critical condition, transfer notifications of neonatal patients were not usually posted on the Band before the transfer. On the day after the transfer, the professor in charge of the NICU posted a notice of the diagnosis, treatment, and condition of the patient on the Band so that the information was shared with the obstetrician who transferred the patient. The professor also issued daily updates of the condition of any patient who had been transferred to the NICU and was still hospitalized. Training information about neonatal diseases was also provided on the Band, so that the local obstetricians could learn about the diseases and take adequate action when similar situations arise. Information about any potential epidemics was also notified and shared on the Band when they were detected at community care centers or nurseries.

The main operators of the DP Band were the supervising professors and the doctor in charge of the department. Local pediatricians notified the reason for the transfer and condition of the patient on the Band before transferring the patient. A doctor in charge or a professor responded to the local pediatrician about the patient’s condition, diagnosis, and treatment plans in real time. When necessary, a professor or a doctor in charge could also share information about the patient's progress after the diagnosis. In addition, the supervising professors shared information about recent epidemics, the latest treatment guidelines, and any other information that might be useful for the training of local pediatricians in the community. Useful information, about conferences, events, and so on, was also shared on the Band. Pursuant to the Personal Information Protection Act, all personally identifiable information was removed before any information was posted on the NICU Band and DP Band. Our study was approved by the Institutional Review Board of Chonbuk National University Hospital.

### Questionnaire

After running the Chonbuk National University Children's Hospital Bands, a survey was conducted with 51 obstetricians and 90 pediatricians who joined the Bands. The professors and doctors in charge, who ran the patient transfer information system, developed an electronic questionnaire using Google Forms. The questionnaire consisted of 14 questions, spanning 7 pages. Multiple-choice questions were used to query the respondent's department of specialization and sex as well as the duration and frequency of the Band usage. The change in number of patients transferred and time required for transfers was also surveyed to evaluate the effectiveness of the Bands. Satisfaction levels were assessed in 6 categories using Likert scales (5-point scales, with 5 points for very satisfied and 1 point for very dissatisfied) for both categorical satisfaction and overall satisfaction. These 6 categories included information about vacant beds and available equipment in the hospital, information about the transferred patient status, communication with the doctor in charge, rapport with the parents of the patient, decreased time needed for transfer, and checking the diagnosis and confirming the treatment. Short answer questions were used for any additional requests and comments. The survey was tested with the professors and doctors at Chonbuk National University Children's Hospital in the exact same way, as it would be used with local physicians before being conducted with the local physicians. The real closed e-survey was conducted from August 2015 to October 2015. The questionnaire was advertised through the Naver Band and posted on Google Forms. The Web address was sent out to the participants. Only the participants who received the Web address by email and had a Google account could access the site and participate in the survey. The participant entered the site and read the information about the purpose of the survey and how they could participate. Then, they responded to the questions voluntarily. No incentives were offered for participating in the survey. Responses were automatically saved in Google's database. The survey could be submitted once the required questions were answered. Once submitted, the respondent was not allowed to edit or review their responses. The survey was submitted after mandatory questions were answered. Multiple entries from the same individual were not allowed by a built-in function provided by Google Forms. A copy of survey questionnaire can be found in Multimedia Appendix 1.

### Data Analysis

The statistical analysis was conducted with SPSS, version 21 (IBM Corporation., Armonk, NY, USA), using frequency analysis and bipartite logistic regression analysis as statistical test methods, with *P* values of less than .05 indicating statistical significance. Frequency analysis was used for the age and sex of the physicians who joined the Bands as well as frequency of usage, number of patients transferred, and time required for transfers to estimate Band usage. For the analysis of factors related to satisfaction, the survey results were divided into a group of highly satisfied respondents (5 points) and a group of all other respondents (4 points and below). Then, binary logistic regression analysis was performed between the 2 groups. We also performed univariate logistic regression analysis and backward multivariate logistic regression analysis to test the correlation between the factors.

## Results

### Children's Hospital Band Sign-Up and Questionnaire Response

The number of obstetricians in the Jeollabuk-do region who joined the NICU Band was 51 (77% of the total number of obstetricians in the Jeollabuk-do region). Of those, 34 (66%) were male. The number of pediatricians in the Jeollabuk-do region who joined the DP Band was 90 (68% of the total number of pediatricians in the Jeollabuk-do region). Of those, 40 (44%) were male. The questionnaire was answered by 78% (40/51) obstetricians and 63% (57/90) pediatricians. Of the obstetricians who answered the questionnaire, 67% (27/40) were male and 65% (26/40) were aged 40–49 years. Of the pediatricians who answered the questionnaire, 54% (31/57) were male and 51% (29/57) were aged 40–49 years (Table 1).

**Table 1:** Members of the transfer information systems and respondents to the questionnaire.

Factor	Obstetrician in local clinic	Pediatrician in local clinic
Number of physicians who participated in the transfer information system	51	90
Total number of respondents to the questionnaire (%)	40 (78)	57 (63)
Number of male doctors who replied to the survey (%)	27 (67)	31 (54)
Number of members aged between 30 to 39 years (%)	1 (2)	10 (17)
Number of members aged between40 to 49 years (%)	26 (65)	29 (51)
Number of members aged older than 50 years (%)	13 (32)	18 (31)

### Frequency and Effects of Using the Children's Hospital Bands

**Figure 1:**
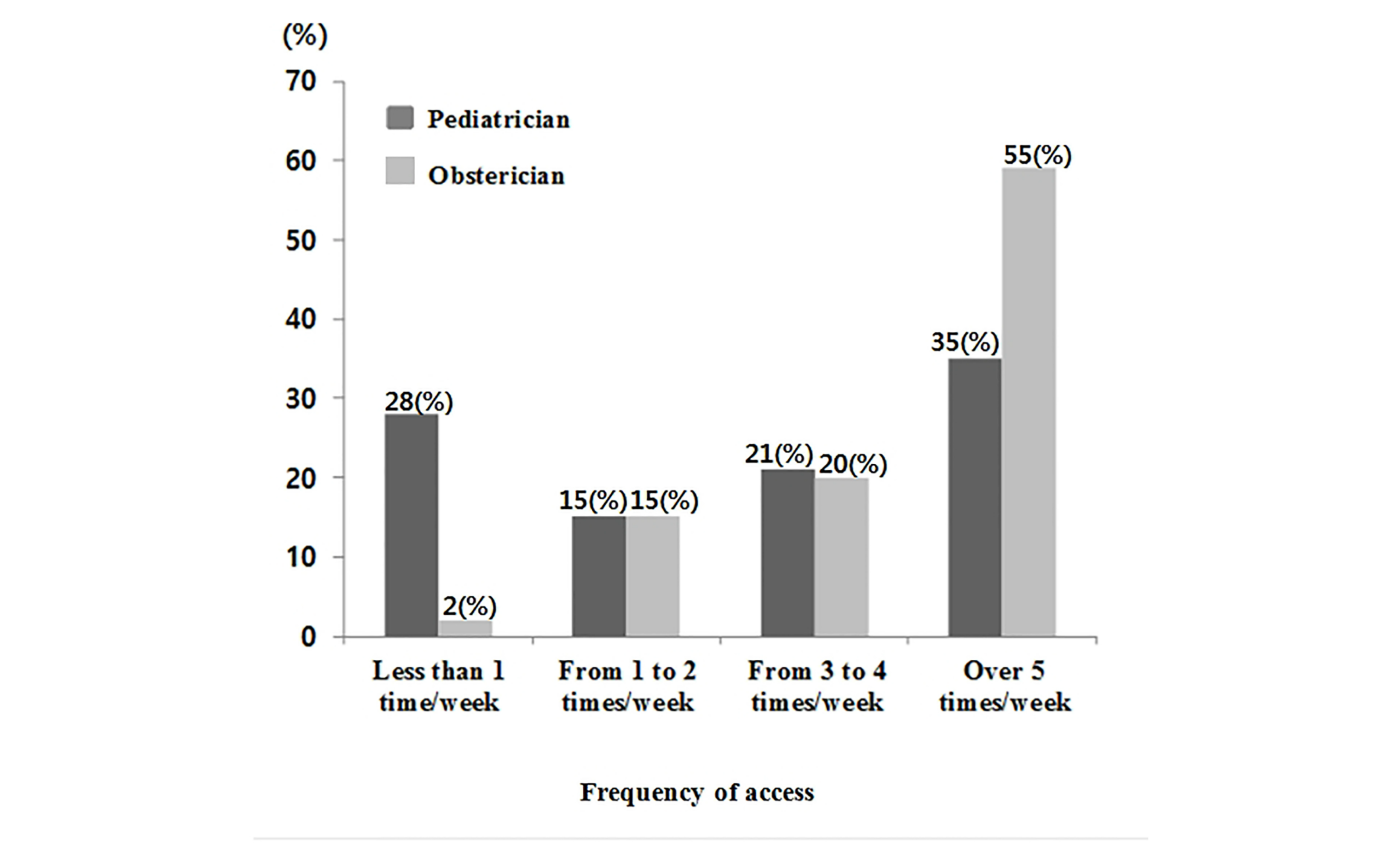
Frequency of access to the patient transfer information systems.

The most common frequency of using the NICU Band, as indicated by 55% (22/40) respondents, was 5 times or more per week. The preferred means of access included using mobile phones by 90% (36/40) respondents and both mobile phones and computers by 10% (4/40) respondents. As for the DP Band, 35% (20/57) respondents used the band 5 times or more per week. The preferred means of access included using mobile phones by 92% (53/57) respondents and both mobile phones and computers by 4% (2/57) respondents (Figure 1). Since using the Children's Hospital Band, 65% (26/40) obstetricians and 40% (23/57) pediatricians responded that the number of patients transferred had increased and 72% (29/40) obstetricians and 59% (34/57) pediatricians responded that the time required for transfers had decreased.

### Factors Related to Satisfaction With Using the Children's Hospital Band

**Figure 2:**
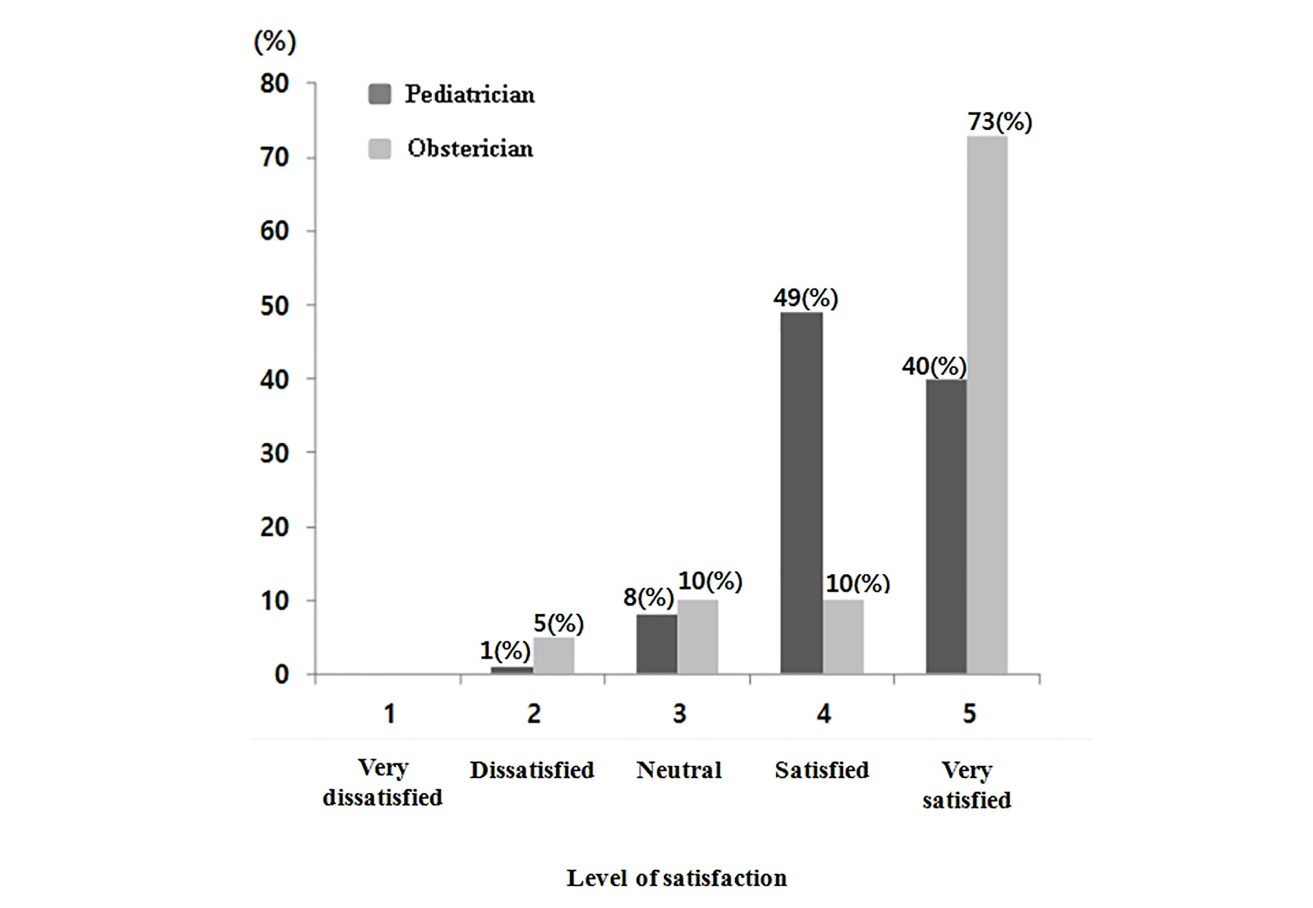
Level of satisfaction with the patient transfer information systems.

In the survey for overall satisfaction with using the Children's Hospital Band, 83% (33/40) obstetricians and 89% (51/57) pediatricians rated it as 4 points or higher (satisfied or very satisfied; Figure 2).

When the factors influencing satisfaction were grouped into 6 categories and the correlation between the factors and satisfaction was tested by univariate regression analysis, the results were statistically significant for both obstetricians and pediatricians (Tables 2 and 3).

**Table 2:** Univariate logistic regression analysis of each factor that affected satisfaction with the transfer information system by obstetricians in the local clinic.

Factor	Odds ratio	95% CI	*P*
Information about vacant beds and available equipment in the hospital	3.6	1.4-8.9	.005
Information about the status of the transferred patient	12.8	1.5-103.7	.02
Communication with the doctor in charge	6.5	1.9-22.3	.003
Rapport with the parent(s) of the patient	6.4	1.5-27.1	.01
Decreased time needed for transfer	5.1	1.7-14.2	.002
Checking the diagnosis and confirming the treatment	5.2	1.7-15.9	.004

**Table 3:** Univariate logistic regression analysis of each factor that affected satisfaction with the transfer information system by pediatricians in the local clinic.

Factor	Odds ratio	95% CI	*P*
Information about vacant beds and available equipment in the hospital	2.5	1.3-4.6	.002
Information about the status of the transferred patient	2.1	1.1-4.3	.02
Communication with the doctor in charge	3.3	1.5-7.2	.002
Rapport with the parent(s) of patient	3.0	1.4-6.5	.004
Decreased time needed for transfer	4.5	1.7-11.7	.002
Checking the diagnosis and confirming the treatment	6.6	2.2-19.7	.001

To identify which of the factors were most strongly correlated with high satisfaction (5 points), backward multivariate bipartite logistic regression analysis was performed between the group with the satisfaction rating of 5 points and the group with the satisfaction rating of 4 points and below, with the data corrected for sex and age. For obstetricians, the ability to communicate with doctors in charge (odds ratio 29, 95% CI 1.311-674.4, *P*=.03) and reduction in time required for transfers (odds ratio 6.5, 95% CI 1.304-37.1, *P*=.02) were highly correlated with satisfaction. For pediatricians, the ability to check the diagnosis and treatment of the patients transferred (odds ratio 3.6, 95% CI 1.276-10.164, *P*=.01) and reduction in time required for transfers (odds ratio 5.6, 95% CI 1.598-19.65, *P*=.07) were highly correlated with satisfaction (Table 4).

**Table 4:** Adjusted multivariate logistic regression analysis of factors that influence satisfaction with the transfer information systems.

	Factor	Odds ratio	95% CI	*P*
**Obstetrician of local clinic**				
	Communication with the doctor in charge	29.7	1.3-674.4	.03
	Decreased time needed for transfer	6.5	1.3-37.1	.02
**Pediatrician of local clinic**				
	To check the diagnosis and confirm the treatment	3.6	1.2-10.1	.01
	Decreased time needed for transfer	5.6	1.5-19.6	.007

### Additional Demands and Comments Regarding the Children's Hospital Band

Regarding the additional improvements and developments that the local physicians would like to see in the Children's Hospital Band, 52% (21/40) obstetricians mentioned the need to expand coverage of the Children's Hospital Band to other regions and 25% (10/40) obstetricians mentioned the need for real-time monitoring of hospital beds available for transfers. On the other hand, 49% (28/57) pediatricians mentioned the need for real-time monitoring of hospital beds available for transfers, 29% (17/57) pediatricians mentioned faster responses concerning diagnosis and treatment of transferred patients, and 28% (16/57) pediatricians mentioned concerns over the possible leaking of patient information.

One of the obstetricians expressed the difficulty of patient transfers before using the Children's Hospital Band by saying,

Honestly, as an obstetrician, I would do everything to avoid the transfer process altogether. It was definitely not a pleasant experience getting on the phone and speaking as if I had done something wrong.

One obstetrician commented on the benefit of using the Children's Hospital Band by saying,

Using the Band helps the transfer process a lot. I can now focus on the delivery with greater peace of mind.

One pediatrician also commented,

I am satisfied with the Band because the response I get about the condition of transferred patients is faster than by paper.

Other comments included,

The Band should be expanded to include pediatric surgery, pediatric orthopedics, and many other departments.

and

In addition to the better patient transfer experience, I am also satisfied with other features of the Band, such as information about recent epidemics and refresher training on diseases.

## Discussion

### Principal Findings

A social media platform was used to run neonatal and pediatric patient transfer information systems to facilitate communication between Chonbuk National University Children's Hospital and local obstetricians and pediatricians in the Jeollabuk-do region. Analysis of the survey responses from the local physicians showed that the users were highly satisfied. Although each group reported different satisfaction factors and additional demands, both groups saw increased numbers of transferred patients and reductions in time required for the transfers since the transfer system was introduced.

The local physicians were highly satisfied with the Chonbuk National University Children's Hospital Bands as they provided real-time updates on bed information of the regional university hospital and allowed communication about the patients’ medical information. Factors associated with satisfaction with the Children's Hospital Bands varied between the obstetricians and pediatricians; the obstetricians' main factor of satisfaction was the ability to communicate with the doctors in charge, whereas the pediatricians regarded the ability to check the diagnosis and treatment information of transferred patients as the most important factor of satisfaction. The difference in satisfaction factors between the obstetricians and pediatricians can be explained in the types of patients transferred. Many of the patients transferred from local obstetric clinics and hospitals are high-risk newborn babies with emergencies occurring immediately after the delivery. In dealing with such patients in need of immediate attention with potentially serious outcomes in survivability and medical disputes, the local physicians regard the ability to identify available hospital beds and communicating with doctors in charge of utmost importance [14]. On the other hand, local pediatric clinics and hospitals tend to transfer patients not for emergency measures but for more advanced diagnosis and treatment [15,16].

Therefore, the varying needs and satisfaction factors of each user group suggest that a customized transfer system is required for each field. Satisfaction with the Children's Hospital Bands can be summarized as the reduction in time required for patient transfers, information sharing, and mutual communication, which is made possible through easy access and provision of information about available hospital beds. Delivering the system on a social media platform can overcome the limitations of existing systems that provide information in one direction only.

### Comparison With Prior Work

In previous studies of patient transfer systems, Shin (2007) reported the necessity of establishing region-specific health care systems and transfer systems for South Korea by benchmarking the neonatal patient transfer systems of advanced countries [17], whereas Chang (2011) suggested that the establishment of adequately regionalized patient transfer systems was necessary for efficient neonatal intensive care [1]. Even before the popularization of social media, state-initiated patient transfer systems based on mail, telephone, and the personal computer-era Internet were used in numerous countries, but many were regarded as inefficient. In contrast, the running of our regional patient transfer information system on a social media platform proved to be highly satisfying among local physicians. As suggested in previous studies, various efforts should be made to improve satisfaction with the implementation of transfer systems for the efficient treatment of seriously ill neonatal patients, such as improving accessibility to such transfer information systems and adequately identifying the users’ needs, as well as through sufficient leveling of hospitals, regionalization, and the introduction of inter-regional transport systems by reforming the facilities, equipment, and structures.

### Limitations

This study has a few limitations. First, the survey was conducted with local physicians who are associated with a single university hospital. Therefore, it would be difficult to generalize the survey results to other regions and other hospitals. In case of Japan, the neonatal patient transfer system assigns a level to each NICU and provides a comprehensive view of hospitals available for transfer [18-20]. However, the Chonbuk National University Children's Hospital Bands were limited to providing information about one hospital only. Efforts could be made to benchmark the Japanese transfer system and group multiple hospitals together in each region for a much more effective system. Second, the Children's Hospital Bands only served the obstetricians and pediatricians in the Jeollabuk-do region who joined the Bands; the survey was conducted with those physicians only. Third, the time required for patient transfers in the survey was based on the physician's perception and not objective measurements. For accurate assessment of actual time reduction, objective remeasurements would be necessary.

### Conclusions

In conclusion, the survey of a social media–based patient transfer information system showed that the users were highly satisfied with the provision of information and facilitation of mutual communication, which is necessary for efficient patient transfers. User needs varied depending on the specificity of the patients transferred. In future developments of patient transfer information systems, the various needs for accessibility to the information system and mutual communication should be accommodated adequately in addition to regionalization and appropriate leveling of hospitals. Furthermore, the patient transfer information system used in our study covered one specific region of a country. A highly effective patient transfer information system would need to go beyond the boundaries of a single region; it needs to connect with other regions and, eventually, connect the whole country. For this reason, we suggest that future studies of patient transfer information systems focus on systems that connect one region to another and on systems that cover the whole nation.

## Multimedia Appendix 1

Survey questionnaire.

## Abbreviations

DP Band: Department of Pediatrics Band
NICU Band: Neonatal Intensive Care Unit Band
