# Supplementary material for: Satisfaction Levels and Factors Influencing Satisfaction With Use of a Social App for Neonatal and Pediatric Patient Transfer Information Systems: A Questionnaire Study Among Doctors
Source: JMIR Med Inform. 2016 Aug 4;4(3):e26. doi: 10.2196/medinform.5984 (PMC4990714; doi:10.2196/medinform.5984)
Supplement: Supplementary file 1 [file medinform_v4i3e26_app1.pptm]

## Slide 1
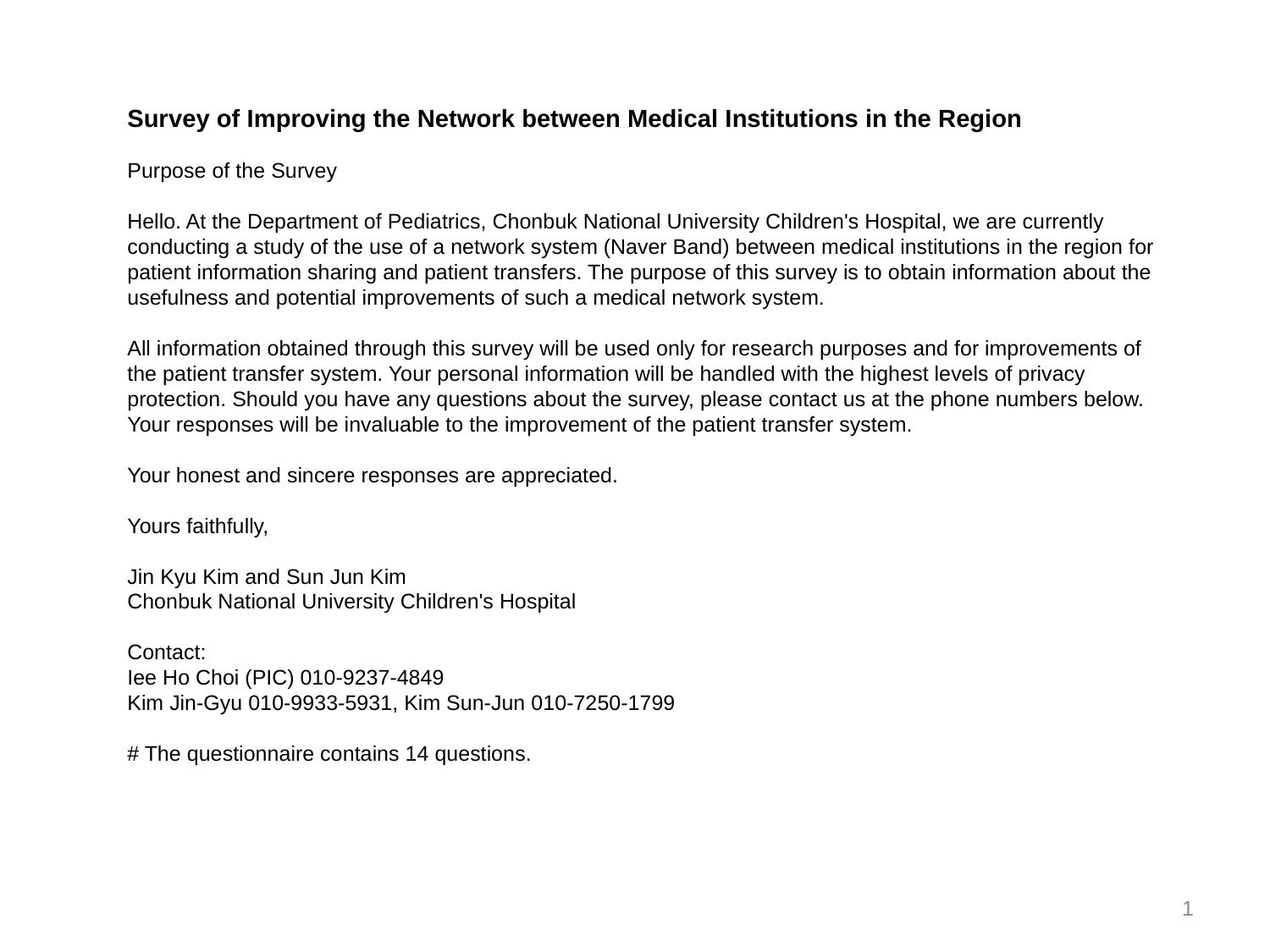

Survey of Improving the Network between Medical Institutions in the Region
Purpose of the Survey
Hello. At the Department of Pediatrics, Chonbuk National University Children's Hospital, we are currently conducting a study of the use of a network system (Naver Band) between medical institutions in the region for patient information sharing and patient transfers. The purpose of this survey is to obtain information about the usefulness and potential improvements of such a medical network system.
All information obtained through this survey will be used only for research purposes and for improvements of the patient transfer system. Your personal information will be handled with the highest levels of privacy protection. Should you have any questions about the survey, please contact us at the phone numbers below. Your responses will be invaluable to the improvement of the patient transfer system.
Your honest and sincere responses are appreciated.
Yours faithfully,
Jin Kyu Kim and Sun Jun Kim
Chonbuk National University Children's Hospital
Contact:
Iee Ho Choi (PIC) 010-9237-4849
Kim Jin-Gyu 010-9933-5931, Kim Sun-Jun 010-7250-1799
# The questionnaire contains 14 questions.
1

## Slide 2
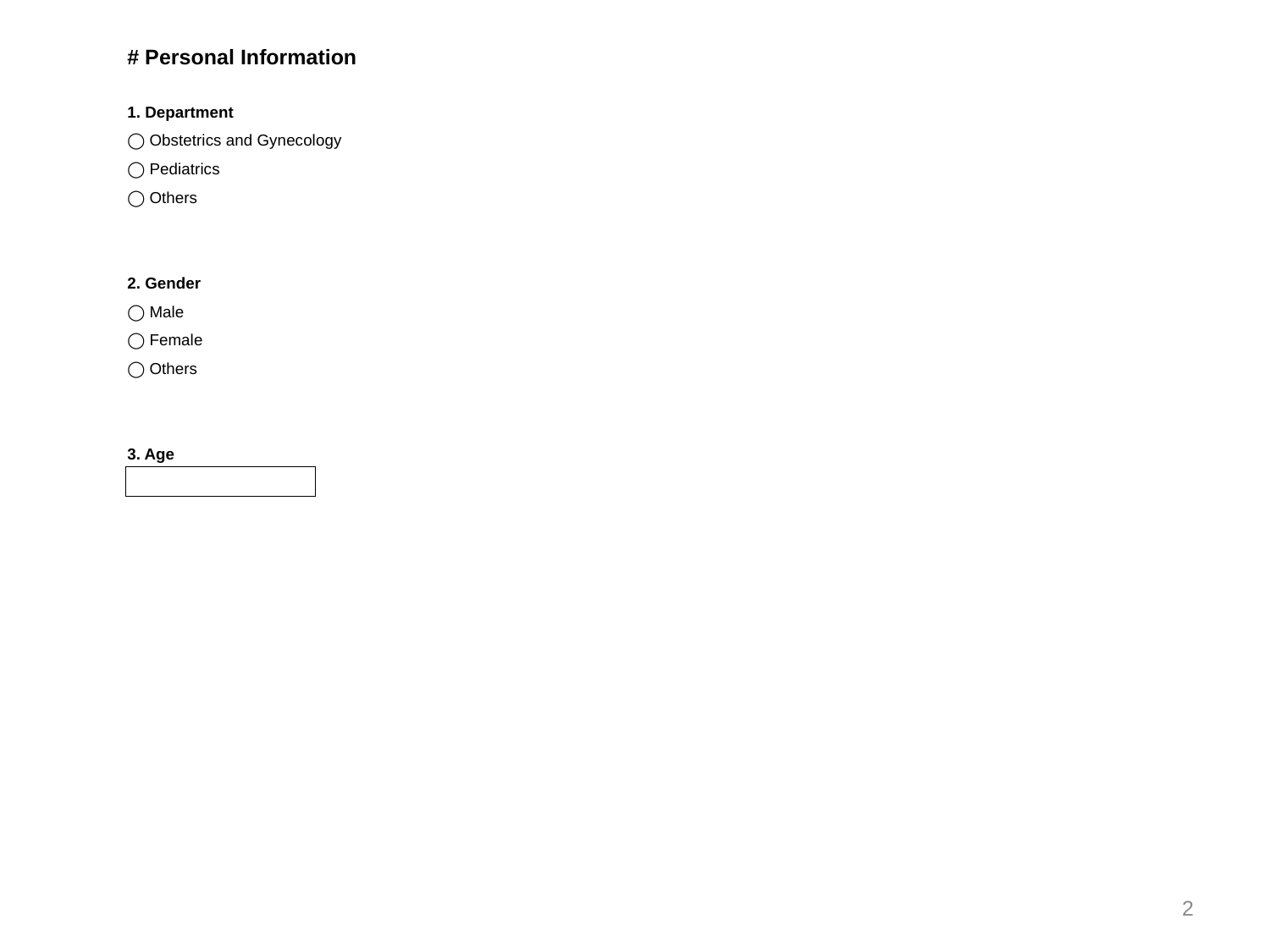

# Personal Information
1. Department
◯ Obstetrics and Gynecology
◯ Pediatrics
◯ Others
2. Gender
◯ Male
◯ Female
◯ Others
3. Age
2

## Slide 3
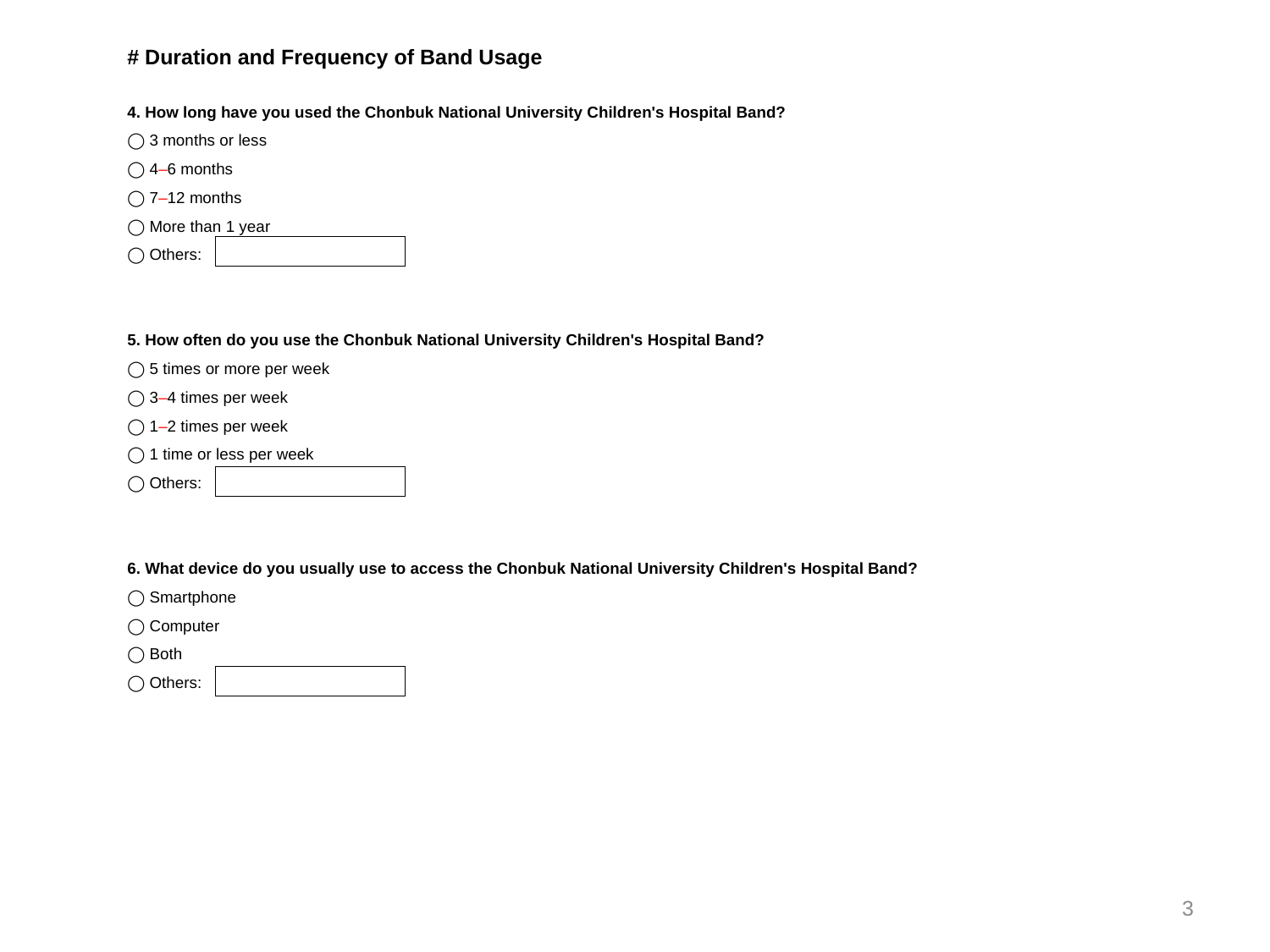

# Duration and Frequency of Band Usage
4. How long have you used the Chonbuk National University Children's Hospital Band?
◯ 3 months or less
◯ 4–6 months
◯ 7–12 months
◯ More than 1 year
◯ Others:
5. How often do you use the Chonbuk National University Children's Hospital Band?
◯ 5 times or more per week
◯ 3–4 times per week
◯ 1–2 times per week
◯ 1 time or less per week
◯ Others:
6. What device do you usually use to access the Chonbuk National University Children's Hospital Band?
◯ Smartphone
◯ Computer
◯ Both
◯ Others:
3

## Slide 4
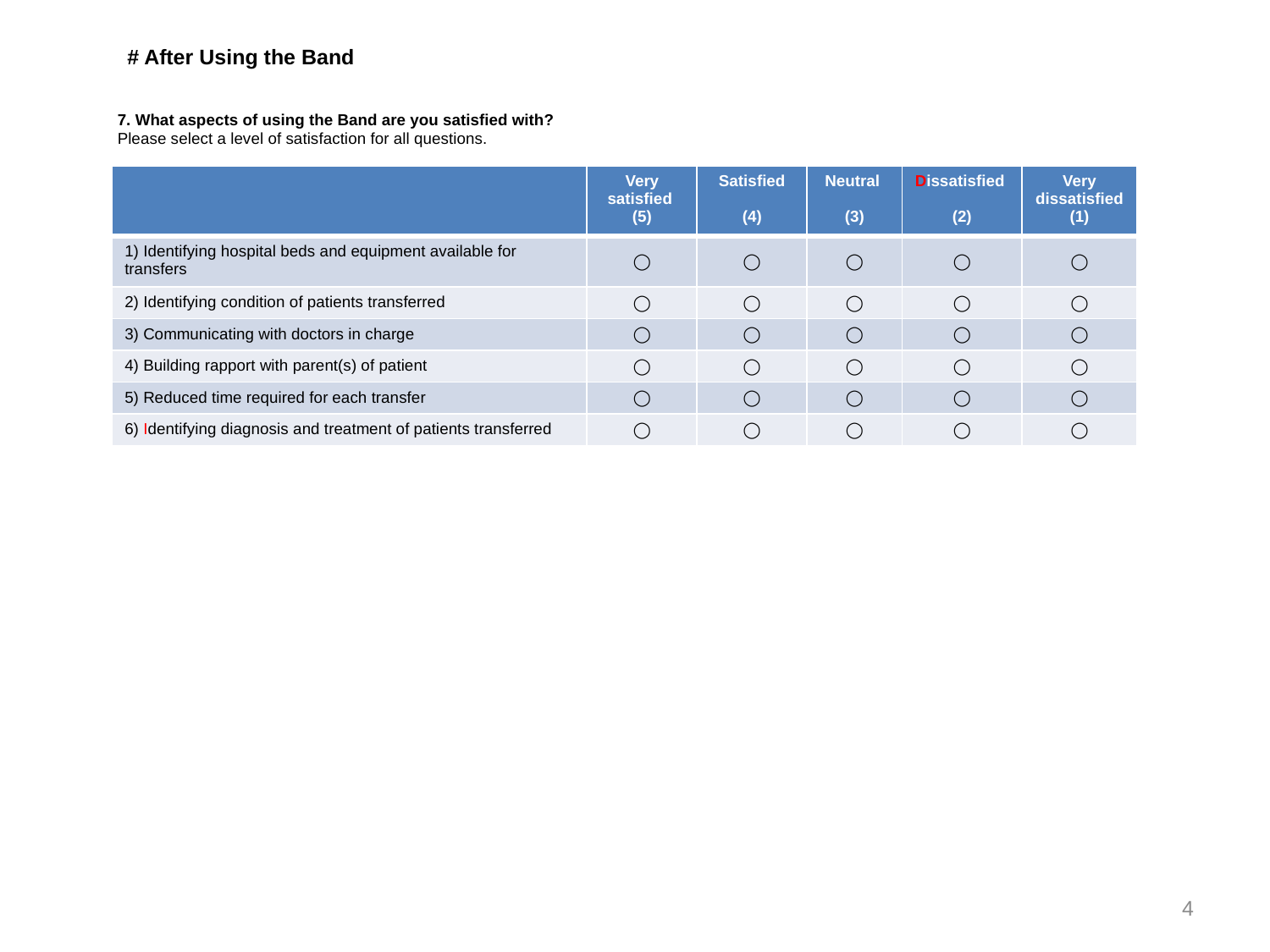

# After Using the Band
7. What aspects of using the Band are you satisfied with?
Please select a level of satisfaction for all questions.
| | Very satisfied (5) | Satisfied (4) | Neutral (3) | Dissatisfied (2) | Very dissatisfied (1) |
| --- | --- | --- | --- | --- | --- |
| 1) Identifying hospital beds and equipment available for transfers | ◯ | ◯ | ◯ | ◯ | ◯ |
| 2) Identifying condition of patients transferred | ◯ | ◯ | ◯ | ◯ | ◯ |
| 3) Communicating with doctors in charge | ◯ | ◯ | ◯ | ◯ | ◯ |
| 4) Building rapport with parent(s) of patient | ◯ | ◯ | ◯ | ◯ | ◯ |
| 5) Reduced time required for each transfer | ◯ | ◯ | ◯ | ◯ | ◯ |
| 6) Identifying diagnosis and treatment of patients transferred | ◯ | ◯ | ◯ | ◯ | ◯ |
4

## Slide 5
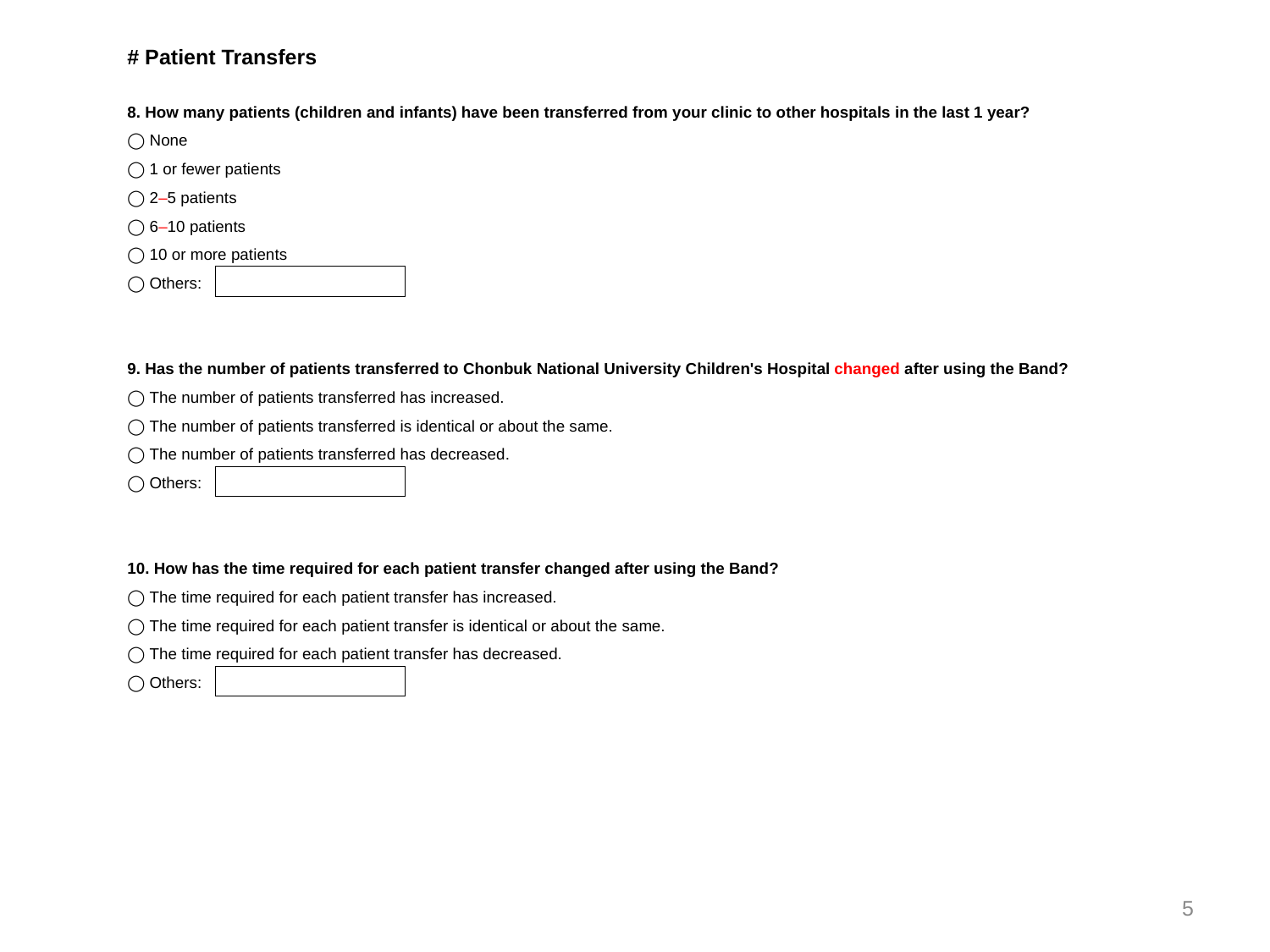

# Patient Transfers
8. How many patients (children and infants) have been transferred from your clinic to other hospitals in the last 1 year?
◯ None
◯ 1 or fewer patients
◯ 2–5 patients
◯ 6–10 patients
◯ 10 or more patients
◯ Others:
9. Has the number of patients transferred to Chonbuk National University Children's Hospital changed after using the Band?
◯ The number of patients transferred has increased.
◯ The number of patients transferred is identical or about the same.
◯ The number of patients transferred has decreased.
◯ Others:
10. How has the time required for each patient transfer changed after using the Band?
◯ The time required for each patient transfer has increased.
◯ The time required for each patient transfer is identical or about the same.
◯ The time required for each patient transfer has decreased.
◯ Others:
5

## Slide 6
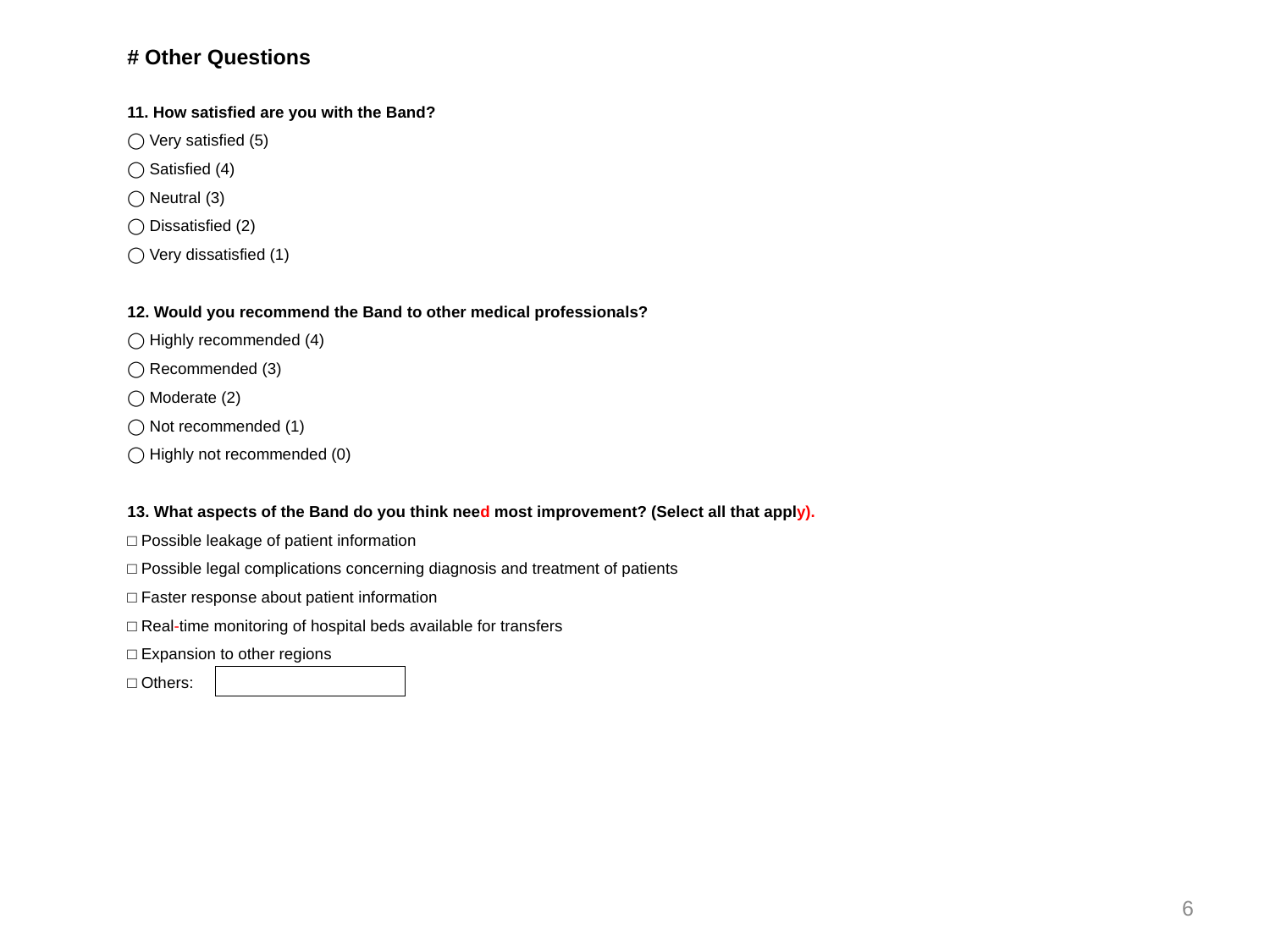

# Other Questions
11. How satisfied are you with the Band?
◯ Very satisfied (5)
◯ Satisfied (4)
◯ Neutral (3)
◯ Dissatisfied (2)
◯ Very dissatisfied (1)
12. Would you recommend the Band to other medical professionals?
◯ Highly recommended (4)
◯ Recommended (3)
◯ Moderate (2)
◯ Not recommended (1)
◯ Highly not recommended (0)
13. What aspects of the Band do you think need most improvement? (Select all that apply).
□ Possible leakage of patient information
□ Possible legal complications concerning diagnosis and treatment of patients
□ Faster response about patient information
□ Real-time monitoring of hospital beds available for transfers
□ Expansion to other regions
□ Others:
6

## Slide 7
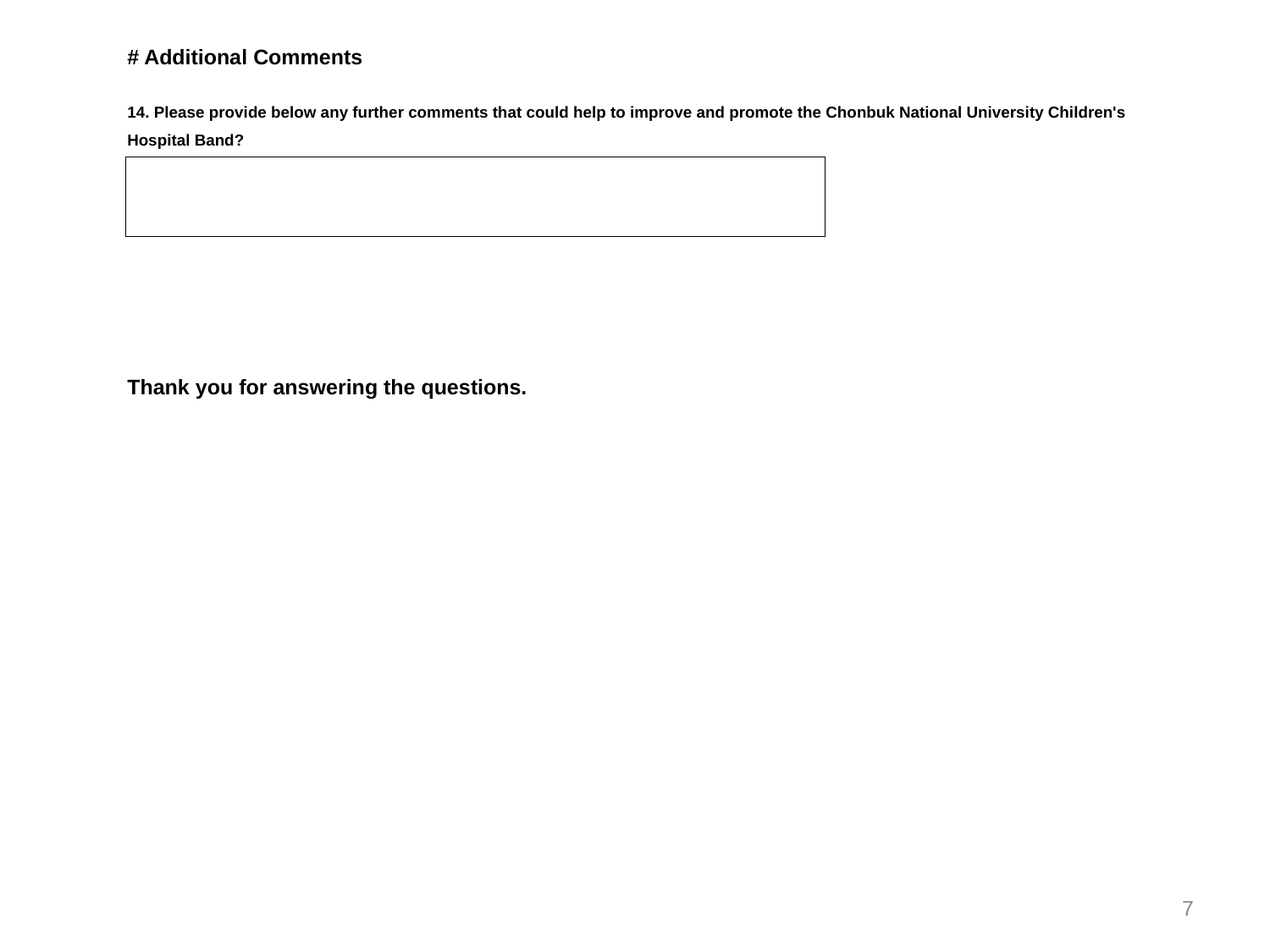

# Additional Comments
14. Please provide below any further comments that could help to improve and promote the Chonbuk National University Children's Hospital Band?
Thank you for answering the questions.
7
